# Comparative Genotyping of *Campylobacter jejuni* Strains from Patients with Guillain-Barré Syndrome in Bangladesh

**DOI:** 10.1371/journal.pone.0007257

**Published:** 2009-09-30

**Authors:** Zhahirul Islam, Alex van Belkum, Jaap A. Wagenaar, Alison J. Cody, Albert G. de Boer, Helen Tabor, Bart C. Jacobs, Kaisar A. Talukder, Hubert P. Endtz

**Affiliations:** 1 International Centre for Diarrheal Diseases Research, Dhaka, Bangladesh; 2 Erasmus MC, Rotterdam, The Netherlands; 3 Central Veterinary Institute of Wageningen UR, Lelystad, The Netherlands; 4 Faculty of Veterinary Medicine, Utrecht University, Utrecht, The Netherlands; 5 Department of Zoology, University of Oxford, Oxford, United Kingdom; 6 National Microbiology Laboratory, Canadian Science Centre for Human and Animal Health, LCDC, Winnipeg, Canada; Charité-Universitätsmedizin Berlin, Germany

## Abstract

**Background:**

*Campylobacter jejuni* is a common cause of acute gastroenteritis and is associated with post-infectious neuropathies such as the Guillain-Barré syndrome (GBS) and the Miller Fisher syndrome (MFS). We here present comparative genotyping of 49 *C. jejuni* strains from Bangladesh that were recovered from patients with enteritis or GBS. All strains were serotyped and analyzed by lipo-oligosaccharide (LOS) genotyping, amplified fragment length polymorphism (AFLP) analysis, multilocus sequence typing (MLST), and pulsed-field gel electrophoresis (PFGE).

**Methodology/Principal Findings:**

*C. jejuni* HS:23 was a predominant serotype among GBS patients (50%), and no specific serotype was significantly associated with GBS compared to enteritis. PCR screening showed that 38/49 (78%) of strains could be assigned to LOS classes A, B, C, or E. The class A locus (4/7 vs 3/39; p<0.01) was significantly associated in the GBS-related strains as compared to enteritis strains. All GBS/oculomotor related strains contained the class B locus; which was also detected in 46% of control strains. Overlapping clonal groups were defined by MLST, AFLP and PFGE for strains from patients with gastroenteritis and GBS. MLST defined 22 sequence types (STs) and 7 clonal complexes including 7 STs not previously identified (ST-3742, ST-3741, ST-3743, ST-3748, ST-3968, ST-3969 and ST-3970). *C. jejuni* HS:23 strains from patients with GBS or enteritis were clonal and all strains belonged to ST-403 complex. Concordance between LOS class B and ST-403 complex was revealed. AFLP defined 25 different types at 90% similarity. The predominant AFLP type AF-20 coincided with the *C. jejuni* HS:23 and ST-403 complex.

**Conclusion/Significance:**

LOS genotyping, MLST, AFLP and PFGE helped to identify the HS:23 strains from GBS or enteritis patients as clonal. Overall, genotypes exclusive for enteritis or for GBS-related strains were not obtained although LOS class A was significantly associated with GBS strains. Particularly, the presence of a clonal and putative neuropathogenic *C. jejuni* HS:23 serotype may contribute to the high prevalence of *C. jejuni* related GBS in Bangladesh.

## Introduction

*Campylobacter jejuni* is the most significant bacterial cause of human gastroenteritis [1], [2], [3], [4]. Clinical syndromes vary from mild to severe and from gastroenteritis to extraintestinal diseases. This latter category includes acute autoimmune neuromuscular complications such as the Guillain-Barré syndrome (GBS) and Miller-Fisher syndrome [5]. The pathogenesis of Campylobacter-induced GBS is complex and involves host susceptibility factors as well as bacterial virulence factors [6], [7], [8]. The development of these autoimmune neuropathies after *C. jejuni* infection is thought to be primarily related to sialylated lipooligosaccharides (LOS) on the cell surface of *C. jejuni*. These exhibit significant molecular mimicry with gangliosides on human peripheral nerves [9], [10], [11], [12], [13]. Most patients who develop GBS after *C. jejuni* enteritis have IgG autoantibodies that react with gangliosides (such as GM1, GD1a, and GQ1b) [14]. Comparison of the LOS loci of various *C. jejuni* strains has demonstrated that only the class A, B and C LOS loci contain the genes that are necessary for the biosynthesis of ganglioside mimics [15].

Extensive effort has been put into the identification of novel determinants of *C. jejuni* associated with the development of GBS [16], [17]. In Japan, South Africa, China, and Mexico, *Campylobacter* strains with specific Penner heat-stabile (HS) serotypes, including HS:19 and HS:41, were overrepresented among strains isolated from GBS patients [18], [5], [19]. *C. jejuni* HS:19 and HS:41 are clonal which suggests that these serotypes may have unique and specific virulence properties that trigger GBS [20]. However, more recent data has shown that these neuropathogenic properties are not restricted to specific HS serotypes as other serotypes commonly isolated from enteritis patients (HS:1, HS:2, and HS:4 complex) are also found in patients with GBS [21]. We recently reported non-HS:19 and non-HS-41 *C. jejuni* serotypes that are overrepresented among strains from GBS patients in Bangladesh [22]. Recently, we reported a high frequency of Campylobacter-related GBS from Bangladesh (12^th^ ASCODD).

The aim of the present study was to investigate the genetic heterogeneity of *C. jejuni* strains isolated from GBS and enteritis patients between 2006 and 2007 in Bangladesh. In this comparative genomic analysis, multi-locus sequence typing (MLST), amplified fragment length polymorphism (AFLP), LOS class PCR typing, and pulsed-field gel electrophoresis (PFGE) were employed to define detailed strain specific genotypes.

## Materials and Methods

### Bacterial strains

A systematic hospital-based study has been carried out among GBS patients in Dhaka, Bangladesh between 2006 and 2007. During this period, we isolated 10 *C. jejuni* strains from stool specimens of GBS patients and 39 *C. jejuni* from enteritis patients [22]. All GBS patients fulfilled the diagnostic of GBS criteria [23]. Bacteria were grown on blood agar plates with 5% sheep blood, at 37°C for 48 h under micro-aerobic conditions, with 6% O_2_, 7% CO_2_, 80% N_2_, and 7% H_2_ using the Anoxomat system (Anoxomat^TM^ Mart II, Drachten, The Netherlands). Bacteria were stored at −80°C in 15% glycerol in brain heart infusion broth. All strains were serotyped with the heat-stable (HS) serotyping schemes of Penner at the National Laboratory for Enteric Pathogens, Canadian Science Centre for Human and Animal Health, Winnipeg, Canada [24]. The project protocol was reviewed and approved by the institutional review board and the ethical committees at the Dhaka Medical College and Hospital, Dhaka, Bangladesh. Verbal informed consent was obtained from all patients and controls.

### Bacterial DNA isolation

Genomic DNA was isolated with the Qiagen Genomic DNA purification kit according to the manufacturer's instructions (Qiagen, Venlo, The Netherlands).

### Determination of the LOS locus class

To determine the LOS class in *C. jejuni* strains, we used specific primer sets for the classes A/B, C, D, and E, based on the DNA sequence of genes unique to the respective LOS locus class(es) described earlier [9]. To discern between classes A and B, we used a primer set that was based on the DNA sequence of *orf5-II* [9]. PCR assays were performed using a Thermocycler 60 (Biomed GmbH) with a program consisting of 40 cycles of 1 minute at 94°C, 1 minute at 52°C, and 2 minutes at 74°C. Per reaction, approximately 50 ng of template DNA was used in a buffer system consisting of 10 mM Tris-HCl (pH 9.0), 50 mM KCl, 1.5 mM MgCl_2_, 0.01% gelatin, 0.1% Triton X-100, 0.2 mM of each of the deoxyribonucleotide triphosphates (Promega Corp.), and 0.2 U Super Taq polymerase (HT Biotechnology Ltd.).

### Multilocus sequence typing of *C. jejuni* strains

Nucleotide sequence analysis of internal fragments of seven housekeeping genes (aspartase A, *aspA*; glutamine synthetase, *glnA*; citrate synthase, *gltA*; serine hydroxymethyl transferase, *glyA*; phosphoglucomutase, *pgm*; transketolase, *tkt* and ATP synthase α subunit, *uncA*) was performed as described by Dingle et al. [25]. Where no amplification product was observed on agarose gel electrophoresis, the reaction was repeated substituting primers described by Miller et al. [26]. The same primers used to obtain each amplicon were used for nucleotide sequencing, which was carried out at least once on each DNA strand using BigDye^TM^ Ready Reaction Mix (Version 3, Applied Biosystems, Foster City, CA) at a concentration of ^1^/_32_ of that described in the manufacturer's instructions. Existing and new alleles, sequence types (ST) and clonal complexes were assigned using the MLST database located at http://pubmlst.org/campylobacter/). Sequence types (STs) were assigned to clonal complexes as described by Dingle et al. [25] by identification of central genotypes and the assignment of variants that differed at one, two, or three loci [25], [27]. The data were used to draw an unweighted pair group mean average (UPGMA) dendrogram by using the program START (http://outbreak.ceid.ox.ac.uk/software.htm) [28].

### AFLP analysis and data processing

Strains were typed by AFLP [29]. In short, chromosomal DNA was digested with *Hin*dIII and *Hha*I and simultaneously ligated with restriction site-specific adapters for 2 h at 37°C. This was followed by a preselective PCR using adapter-specific primers with *Hin*dIII (5′-GACTGCGTACCAGCTT) and *Hha*I (5′-GATGAGTCCTGATCGC-3′). Next, an aliquot was subjected to a selective PCR using a fluorescently labelled *Hin*dIII primer that contained an additional A nucleotide at the 3′ end (5′-GACTGCGTACCAGC TTA) and a *Hha*I primer with an A extension (59-GATGAGTCCTGATCGCA). The final products were run on a 7.3% denaturing acrylamide gel for 5 h using a ABI 373A automated DNA sequencer. Fingerprints were collected by fluorography and interpreted with ABI Genescan software (PE Applied Biosystems). Gels were normalized using an internal ROX-labeled size standard included in each lane. Densitometric curves were processed with the GelCompar version 4.1 software (Applied Maths, Kortrijk, Belgium). After normalization and background subtraction, the levels of genetic similarity between AFLP patterns were calculated with the Pearson product-moment correlation coefficient (*r*).

### PFGE

PFGE was performed as previously described [30]. In short, samples of genomic DNA extracted from overnight cultures of the strains were digested with *Sma*I (Boehringer GmbH, Mannheim, Germany). Electrophoresis was performed in 1% SeaKem agarose in 0.53 Tris-borate-EDTA buffer by using a Bio-Rad CHEF Mapper programmed in the auto-algorithm mode (run time, 18 h; switch time, 6.76 to 35.38 s). Gels were stained with ethidium bromide for 30 min, destained in distilled water for 1 h; images of ethidium bromide-stained gels were captured under UV illumination by a video system (Gel DOC 1000; Bio-Rad).

### Data analysis

Electrophoretic patterns from PFGE were compared by means of BioNumerics, version 4.01 (Applied Maths, Sint-Martens-Latem, Belgium). Analysis was based on band position and derived by the Dice coefficient with a maximum position tolerance of 1%. Strains were clustered by the unweighted pair group method using arithmetic averages. Statistical analysis was performed with EpiInfo (version 3.0) using 2×2 contingency tables. Fisher's exact tests were executed and 2-sided *P* values determined. Associations were considered significant at *P*<0.05.

## Results

### Serotyping

Serotyping of the 10 GBS-related strains revealed 4 different HS serotypes [22]. *C. jejuni* HS:19 was encountered in 2/10 (20%) patients. *C. jejuni* HS:23 was found in 5/10 (50%), a predominant serotype in GBS patients. Serotyping of enteritis strains revealed 15 HS-serotypes (Table 1). *C. jejuni* HS:23 was predominant serotype (28%) among enteritis strains.

**Table 1:** MLST analysis of the *C. jejuni* strains from GBS and enteritis patients from Bangladesh.

Strains	Year	Disease^a^	LOS^b^ Class	Penner type(s)^c^		Allele number^e^							
					ST^d^	*aspA*	*glnA*	*gltA*	*glyA*	*pgm*	*Tkt*	*uncA*	CC^f^
BD-07	2006	GBS	A	HS:19	22	1	3	6	4	3	3	3	22
BD-10	2006	GBS	B	HS:23	3219	10	27	33	19	10	5	7	403
BD-22	2006	GBS	B	HS:23	3219	10	27	33	19	10	5	7	403
BD-27	2006	GBS	A	UT	587	1	2	42	4	90	25	8	362
BD-34	2006	GBS	B	HS:23	3219	10	27	33	19	10	5	7	403
BD-39	2006	GBS	A	HS:19	660	1	3	6	4	54	91	3	22
BD-67	2007	GBS	B	HS:23	985	10	27	89	19	10	132	7	403
BD-74	2007	GBS	B	HS:23	3219	10	27	33	19	10	5	7	403
BD-75	2007	GBS	A	HS:55	587	1	2	42	4	90	25	8	362
BD-94	2007	GBS	E	HS:21	2109	4	7	10	4	10	7	1	45
CZ-02	2006	Enteritis	ND	NT	3632	91	2	42	4	169	25	8	UA
CZ-5	2006	Enteritis	ND	HS:15	27	1	2	42	85	11	9	8	362
CZ-9	2006	Enteritis	ND	HS:15	27	1	2	42	85	11	9	8	362
CZ-10	2006	Enteritis	A	HS:41	587	1	2	42	4	90	25	8	362
CZ-12	2007	Enteritis	B	HS:23	3219	10	27	33	19	10	5	7	403
CZ-13	2007	Enteritis	B	HS:23	3219	10	27	33	19	10	5	7	403
CZ-14	2007	Enteritis	B	HS:23	3219	10	27	33	19	10	5	7	403
CZ-16	2007	Enteritis	B	HS:23	3219	10	27	33	19	10	5	7	403
CZ-17	2007	Enteritis	ND	HS:12	3632	91	2	42	4	169	25	8	UA
CZ-19	2007	Enteritis	B	NT	3219	10	27	33	19	10	5	7	403
CZ-20	2007	Enteritis	B	HS:23	3219	10	27	33	19	10	5	7	403
CZ-21	2007	Enteritis	B	HS:23	3219	10	27	33	19	10	5	7	403
CZ-22	2007	Enteritis	B	HS:4	1374	24	2	5	72	2	5	6	UA
CZ-23	2007	Enteritis	ND	NT	3632	91	2	42	4	169	25	8	UA
CZ-26	2007	Enteritis	E	HS:21	2109	4	7	10	4	10	7	1	45
CZ-27	2007	Enteritis	B	HS:23	3219	10	27	33	19	10	5	7	403
CZ-29	2007	Enteritis	B	HS:21	1374	24	2	5	72	2	5	6	UA
CZ-31	2007	Enteritis	B	HS:13	1374	24	2	5	72	2	5	6	UA
CZ-32	2007	Enteritis	C	HS:8	**3968**	8	2	52	68	11	5	7	UA
CZ-33	2007	Enteritis	E	NT	NT								NT
CZ-36	2007	Enteritis	B	HS:53	588	1	82	5	90	2	88	1	UA
CZ-37	2007	Enteritis	E	HS:3,4	**3969**	7	2	33	2	10	3	6	UA
CZ-39	2007	Enteritis	A	HS:10	**3742**	1	**308**	95	49	**436**	**353**	**258**	UA
CZ-46	2007	Enteritis	ND	HS:45	2993	1	2	42	4	11	9	8	362
CZ-51	2007	Enteritis	B	NT	3219	10	27	33	19	10	5	7	403
CZ-54	2007	Enteritis	E	NT	**3970**	4	7	10	249	3	7	1	UA
CZ-57	2007	Enteritis	E	NT	5	7	2	5	2	10	3	6	353
CZ-60	2007	Enteritis	A	HS:10	**3742**	1	**308**	95	49	**436**	**353**	**258**	UA
CZ-69	2007	Enteritis	B	HS:23	985	10	27	89	19	10	132	7	403
CZ-75	2007	Enteritis	ND	HS:45	2993	1	2	42	4	11	9	8	362
CZ-77	2007	Enteritis	ND	UT	**3743**	**233**	2	42	4	90	25	8	UA
CZ-81	2007	Enteritis	B	HS:1	1323	7	17	5	10	11	3	6	353
CZ-85	2007	Enteritis	ND	HS:53	**3741**	**234**	10	2	2	67	12	6	UA
CZ-93	2007	Enteritis	E	HS:53	**3741**	**234**	10	2	2	67	12	6	UA
CZ-94	2007	Enteritis	ND	HS:53	354	8	10	2	2	11	12	6	354
CZ-96	2007	Enteritis	B	HS:40	1377	1	2	42	4	153	9	8	42
CZ-98	2007	Enteritis	ND	HS:53	**3741**	**234**	10	2	2	67	12	6	UA
CZ-99	2007	Enteritis	B	NT	**3748**	**235**	2	42	62	11	9	8	UA
CZ-100	2007	Enteritis	B	HS:66	**3748**	**235**	2	42	62	11	9	8	UA

a GBS, Guillain-Barré syndrome (Islam et al. 2009).

b ND, not belong to LOS class A-E.

c Penner heat-stable (HS) serotypes; UT, untypeable; NT, not typed.

d ST, Sequence type; The MLST ST first reported in this study are indicated in boldface.

e New alleles identified in this study are in boldface.

f UA, unassigned.

### LOS locus class

The results presented in Table 2 indicate that 38/49 (78%) of the *C. jejuni* strains characterized in this study could be assigned to one of the five LOS locus classes (A-E) screened by the class-specific PCR. The class A LOS locus was significantly associated with GBS-associated strains compared to controls strains (57% versus 8%, p<0.01; Table 2). In contrast, the three strains isolated in GBS patients with oculomotor symptoms contained a class B locus. LOS class B was detected in 18/39 (46%) of control strains (Table 2). No LOS locus classes C or D were found in GBS strains. LOS classes C and D were absent in enteritis strains with one exception; strain CZ-32 which had class C. The class E locus was rare in GBS and enteritis (1/10 vs 6/39 respectively).

**Table 2:** LOS biosynthesis loci in *C. jejuni* strains from patients with GBS, and uncomplicated enteritis.

		No. of strains associated with:		
LOS locus class	GBS (n = 7)	GBS/oculomotor (n = 3)	Enteritis (n = 39)	p-value
A (n = 7)	4 (57%)	0	3 (8%)	<0.01^a^
B (n = 23)	2 (28%)	3 (100%)	18 (46%)	0.10^b^
C (n = 1)	0	0	1 (2%)	-
D (n = 0)	0	0	0	-
E (n = 7)	1 (14%)	0	6 (15%)	NS
cUnknown	0	0	11 (28%)	-

a GBS vs enteritis.

b GBS/oculomotor vs enteritis; GBS had oculomotor and ataxia symptom.

c We determined the LOS locus (class A to E); all the GBS related strains used in this study were positive for one of the five identified LOS locus classes; therefore we did not include other LOS classes in this study.

NS, not significant.

### Multilocus sequence typing of *C. jejuni* strains

**Figure 1:**
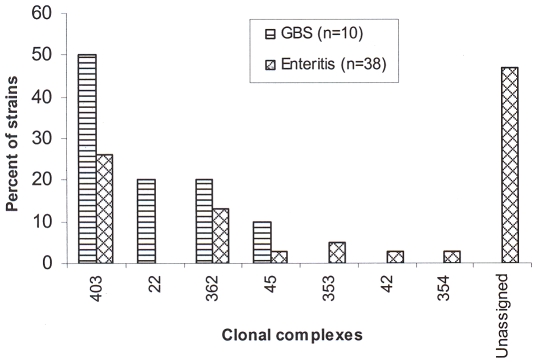
Frequency distribution of *C. jejuni* clonal complexes isolated from GBS and enteritis patients.

**Figure 2:**
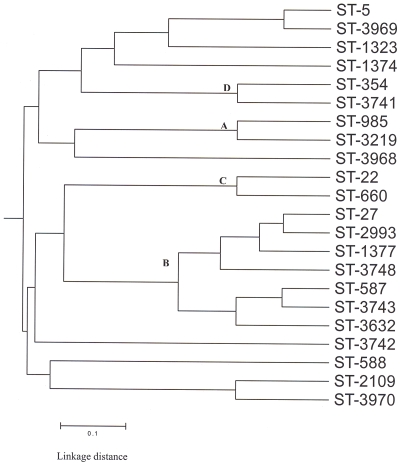
Dendrogram of *Campylobacter jejuni* sequence types, including GBS and enteritis related strains from Bangladesh. The dendrogram was constructed by using UPGMA.

A total of 22 different STs were found belonging to 7 clonal complexes, and a further 18 STs remained unassigned (Table 1). Seven new STs not previously registered in the *Campylobacter* pubMLST database were identified (ST-3742, ST-3741, ST-3743, ST-3748, ST-3968, ST-3969 and ST-3970). The most prevalent lineage was the ST-403 complex (Fig. 1), which included 5 of the 10 (50%) GBS-related strains studied and 10 of the 38 (26%) enteritis strains. The ST-362 complex included 2 of 10 (20%) GBS-related strains studied and 5 of 38 (13%) enteritis strains (Fig. 1). All Penner serotypes HS:23 strains belong to the ST-403 complex. ST-22 and ST-660, which both belong to the ST-22 complex, were found only in GBS-related strains and both strains belong to Penner serotype HS:19 (Table 1). UPGMA clustering of MLST data for *C. jejuni* strains isolated from GBS and enteritis patients yielded 4 major clonal groups (A, B, C and D) consisting 13 STs, and 8 STs were singletons (Fig. 2).

### AFLP

**Figure 3:**
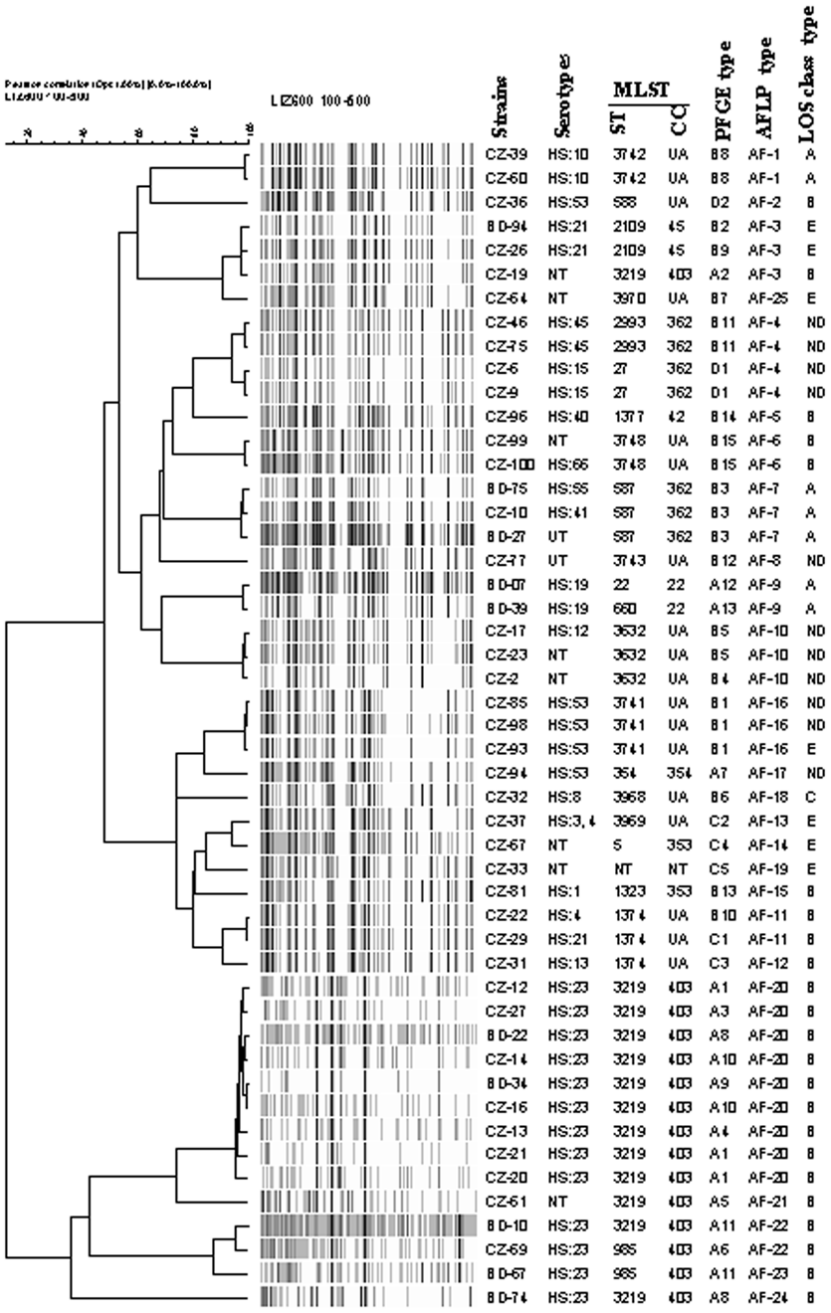
UPGMA dendrogram of AFLP fingerprints from 40 gastroenteritis and 10 strains related to patients with GBS. The percentage of genetic homology between banding patterns is indicated. Serotypes, LOS type, MLST, PFGE and AFLP types are plotted next to dendrogram. UA, unassigned; NT not typed; UT untypeable.

AFLP fingerprints were identified as distinct types when band patterns shared less than 90% similarity according to Duim et al. [29]. Among 49 *C. jejuni* strains, 25 different AFLP types were found. Twelve AFLP types were encountered in more than one strain (Fig. 3). The predominant AFLP type AF-20 was observed in 9 (18%) strains and type AF-3 in 3 (6%) and AF-4 in 4 (8%) strains each (Fig. 3). Seven distinct AFLP fingerprints were found in GBS-related *C. jejuni* strains. The predominant AFLP type AF-20 consisted of two GBS-related strains (BD-22 and BD-34) and 7 enteritis strains; this AFLP type correlated with Penner serotype HS:23 and clonal complex ST-403 (Fig. 3). Strains BD-07 and BD-39 displayed identical fingerprints (AF-9) and belong to the same Penner serotype O:19. AFLP subdivided PFGE type A11 strains (BD-10 and BD-67) into two sub-clusters (AF-22 and AF-23). Twelve AFLP fingerprints of gastroenteritis strains (CZ-31, CZ-32, CZ-33, CZ-36, CZ-51, CZ-54, CZ-77, CZ-81, CZ-94, CZ-96, BD-67 and BD-74) were unique (Fig. 3).

### PFGE

The *SmaI*-PFGE fingerprints contained six to nine bands and resolved into 4 major lineages at 60% similarity [31] (data not shown). These results correlate well with both the MLST clonal complexes and the different sub-clusters as defined by AFLP (Fig. 3). The 4 PFGE lineages could be subdivided in 33 subclusters at 90% similarity (Fig. 3). The majority of the strains belonging to a given clonal complex (ST-403) were also included in the same PFGE group (A). PFGE subdivided AFLP type AF-20 into several subtypes. PFGE analysis of 10 GBS related *C. jejuni* revealed the presence of 7 distinct types. Alhough AFLP and serotyping suggested that BD-07 and BD-39 were clonal (AF-9), these two strains were distinguishable by PFGE (A12 and A13).

### Congruence between typing methods

After cluster analysis of the data obtained by the respective methods and construction of a composite data set comprising the LOS typing, MLST, PFGE and AFLP, a similar clustering of the strains was observed. All ST-403 complex strains belonged to LOS locus class B. The correlation between LOS class B and ST-403 complex was evident in both GBS and enteritis collections (Table 1). Upon analysis of clustering of the sequence types by MLST, 81% showed overlap with AFLP types (Fig. 3).

## Discussion

We performed comparative genomics of a set of 49 *C. jejuni* strains isolated from GBS and enteritis patients in Bangladesh by MLST, AFLP, LOS typing and PFGE fingerprinting. This is the first report on molecular characterization of GBS and enteritis related *C. jejuni* strains from Bangladesh. Cluster analysis of LOS typing, AFLP, PFGE and MLST showed significant overlaps. The LOS class A was significantly over-represented in the GBS-associated strains compared to the enteritis strains. Our MLST analysis demonstrated that all of the Bangladeshi strains with HS:23 serotype are clonal and clearly distinct from the non-HS:23 strains. The clonal complex ST-403 was overlapped by LOS typing, AFLP and PFGE. We recently reported that *C. jejuni* HS:23 serotype is prevalent among GBS and enteritis-related *C. jejuni* strains from Bangladesh [22]. Our comparative genotyping analysis supported that *C. jejuni* HS:23 strains are clonal. However, comparison of a worldwide non-HS:19 associated with GBS and enteritis showed heterogeneity [32].

In the present study, we targeted only five specific classes (A-E) of LOS loci, despite recent increases in the number of LOS locus classes identified [33]. LOS class A, B and C have been associated previously with GBS (9). We identified LOS A or B in 90% of GBS associated strains and in 46% of enteritis strains. Interestingly, we found that the class A locus is significantly associated with GBS without oculomotor symptoms whereas the class B locus associated with GBS with oculomotor symptoms. Previously Nachamkin et al. [34] reported a strong association between GBS-associated *C. jejuni* strains and the simultaneous presence of three LOS biosynthesis genes, *cst-II*, *cgtA* and *cgtB*. Our data confirm these findings, as the combination of *cstII*, *cgtA* and *cgtB* only exist in class LOS A and B. Other studies have demonstrated that the class A, B and C LOS loci contain the specific genes involved in the biosynthesis of ganglioside mimics [9], [15]. Molecular mimicry between Campylobacter LOS and gangliosides in human peripheral nerves is thought to be the mechanism involved in the development of GBS [35].

We have used a variety of molecular techniques to demonstrate the genomic differences or similarities among the *C. jejuni* strains. In this study, we identified 7 distinct clonal complexes with 22 different STs. The most common Bangladeshi lineage was the ST-403 complex (Fig. 2). This predominant clonal complex is corroborated by LOS class B loci. In addition, distribution of STs showed a good concordance between GBS and enteritis related strains (Fig. 2). No representatives of ST-21 were present among GBS and enteritis related strains from Bangladesh, whereas ST-21 is the prevalent complex in the general population structure of *C. jejuni* [25], it is widespread in multiple hosts and has previously been described to be associated with infections in humans, and with livestock and environmental sources; as in chicken, cattle, contaminated milk and water [25], [36]. Molecular epidemiological evidence suggests that this clonal complex is frequently associated with environmental and food borne transmission [36], [37]. Recently, Habib et al. [31] demonstrated that ST-21 complex strongly correlated with class LOS C loci. Both ST-21 and LOS C appear to be rare in Bangladesh. The GBS-associated strains were assigned to different clonal complexes [22], which correlated with earlier data describing heterogeneity among neuropathogenic *C. jejuni* strains [21], [25]). ST-22 and ST-660 (both ST-22 complex) belong to Penner serotype HS:19 and were only found in GBS-related strains; ST-362 complex was the second most prevalent complex found in both GBS and enteritis strains; corroborated with LOS class A or B loci. A number of new STs were identified for the first time in this study (ST-3442, ST-3741, ST-3743, ST-3748, ST-3968, ST-3969 and ST-3970), but were not assigned to any known complex. To date, these unassigned STs have only been found only in enteritis strains from Bangladesh.

Cluster analysis of AFLP data in this study supports previous reports that no distinct subpopulation of *C. jejuni* strains is associated with GBS or enteritis [29]. AFLP analysis revealed that HS:23 strains are clonal but substantial heterogeneity was found among non-HS:23 strains. PFGE and AFLP analysis were shown to have a high level of discriminatory power, although in some cases AFLP was able to distinguish further patterns. In some cases AFLP patterns of the strains were highly similar, whereas PFGE patterns showed differences (Fig. 2). Our PFGE and AFLP data also support those reported in a previous study carried out on Finnish *C. jejuni* strains [38]. The genetic diversity of *C. jejuni* is well recognised and is attributed to a number of distinct phenomena, including genomic rearrangements and horizontal gene transfer [39]. A study carried out in England by Owen et al. [39] showed that *C. jejuni* strains from human strains were highly diverse by PFGE analysis. Previous studies also described that MLST, AFLP, PFGE and DNA microarrays could not identify GBS-specific genetic markers by comparing the genomes of *C. jejuni* strains [16], [21], [25], [29]. Furthermore, no molecular markers specific to GBS were detected after analyzing a highly clonal HS:41 population from South African patients by using a high-throughput AFLP [40]. However, the LOS class typing significantly differentiated GBS-related strains from enteritis in Bangladesh.

In conclusion, our results support *C. jejuni* HS:23 are over represented among GBS related strains in Bangladesh and appear to be clonally related; LOS class A is significantly associated with GBS. The present study revealed a correlation between MLST clonal complex (ST-403) and certain LOS locus class B. Particularly, putative neuropathogenic *C. jejuni* HS:23 serotype may circulate at an elevated prevalence in Bangladesh.
